# Body Roundness Index and Reported Vision Difficulty in U.S. Children and Adolescents: A Cross-Sectional Study of NHANES 2021–2023

**DOI:** 10.3390/healthcare14101352

**Published:** 2026-05-15

**Authors:** Jingwen Wang, Shuai Ouyang, Jia Qu, Ying Li

**Affiliations:** 1 Department of Ophthalmology, Peking Union Medical College Hospital, Chinese Academy of Medical Sciences and Peking Union Medical College, Beijing 100730, China; jingwenwang@student.pumc.edu.cn (J.W.); b2023001111@pumc.edu.cn (S.O.); 2 Research Unit of Myopia Basic Research and Clinical Prevention and Control, Chinese Academy of Medical Sciences (2019RU025), Wenzhou 325027, China; 3 School of Optometry and Ophthalmology and Eye Hospital, Wenzhou Medical University, Wenzhou 325027, China; qujia@eye.ac.cn

**Keywords:** body roundness index, vision difficulty, central adiposity, children and adolescents, NHANES, cross-sectional study

## Abstract

**Background:** Vision difficulty (VD) in childhood and adolescence may affect learning, daily functioning, and quality of life. Although body roundness index (BRI) has emerged as an indicator of central adiposity and cardiometabolic risk, its association with reported VD in pediatric populations remains unclear. **Methods:** We conducted a cross-sectional analysis using data from the National Health and Nutrition Examination Survey (NHANES) August 2021–August 2023. Participants aged 5–17 years with available data on VD, BRI, and covariates were included. VD was defined as self- or proxy-reported difficulty seeing even when wearing glasses or contact lenses. Survey-weighted logistic regression models were used to examine the association between BRI and VD, with progressive adjustment for demographic, socioeconomic, and lifestyle factors. BRI was analyzed as both a continuous variable and weighted quartiles. Restricted cubic spline (RCS) analysis was performed to assess the dose–response relationship. **Results:** A total of 1566 participants were included. In the fully adjusted model, each one-unit increase in BRI was associated with 12% higher odds of VD (odds ratio [OR] = 1.12, 95% confidence interval [CI]: 1.02–1.23, *p* = 0.029). Per 1 standard deviation increase in BRI, the OR for VD was 1.20 (95% CI: 1.03–1.40). Weighted quartile analysis showed imprecise fully adjusted estimates; descriptively, weighted VD prevalence was higher in the highest than in the lowest BRI quartile. RCS analysis showed a significant overall association (*p*-overall = 0.0297) without evidence of non-linearity (*p*-non-linearity = 0.861). **Conclusions:** Higher BRI was associated with greater odds of reported VD among U.S. children and adolescents. Given the cross-sectional design and questionnaire-based outcome, these findings should be interpreted as a population-level association rather than evidence of causality or clinical utility.

## 1. Introduction

Vision plays a central role in child development. During childhood and adolescence, difficulties in seeing may affect academic performance, social participation, daily functioning, and quality of life, with consequences that can extend beyond ocular symptoms themselves [1,2,3]. Because vision difficulty (VD) arising early in life may interfere with learning and psychosocial development, understanding factors associated with VD in pediatric populations is of clinical and public health relevance [4,5].

Previous pediatric vision research has mainly focused on ocular conditions and behavioral exposures, such as refractive error, near work, screen exposure, and limited outdoor time [6,7,8]. In recent years, increasing attention has also been paid to the relationship between systemic health and ocular outcomes [9,10,11]. Excess adiposity, particularly central adiposity, has been associated with chronic low-grade inflammation, insulin resistance, endothelial dysfunction, and microvascular impairment [12,13,14]. These systemic changes may be relevant to ocular tissues and retinal microcirculation, suggesting that adiposity and body fat distribution may be associated with vision-related outcomes [12,13,14,15,16,17].

Body roundness index (BRI), derived from waist circumference and height, was developed to better capture body shape and central fat accumulation than conventional anthropometric indicators [18]. Compared with body mass index (BMI), BRI may better reflect visceral adiposity and the metabolic abnormalities associated with abdominal obesity [19,20]. Higher BRI has been associated with cardiometabolic disorders, including hypertension, dyslipidemia, insulin resistance, and metabolic syndrome [21,22].

Nevertheless, to our knowledge, no prior pediatric population-based study has specifically examined the association between BRI and reported VD. Existing pediatric studies have more often focused on conventional obesity indicators, especially BMI, in relation to myopia, visual impairment, or retinal microvascular changes [15,16,23,24,25,26,27]. By contrast, BRI-related ocular studies have largely examined adolescent myopia or adult retinal disease rather than questionnaire-reported functional VD in children and adolescents [28,29,30].

Reported difficulty seeing despite usual correction captures a functional dimension of vision-related experience that may affect daily activities and perceived visual burden [31,32,33]. In National Health and Nutrition Examination Survey (NHANES), this outcome is assessed by a questionnaire item asking whether participants have difficulty seeing even when wearing glasses or contact lenses. Therefore, the present study focuses on reported VD as a questionnaire-based functional visual complaint, rather than as a measure of visual acuity, refractive status, or diagnosis-specific ocular disease.

Using data from NHANES August 2021–August 2023, the present study investigated the association between BRI and reported VD among U.S. children and adolescents aged 5–17 years. We hypothesized that higher BRI would be associated with greater odds of reported VD and that this association would be approximately linear across the observed range.

## 2. Materials and Methods

### 2.1. Study Design and Study Population

This cross-sectional study used data from NHANES August 2021–August 2023, a nationally representative survey conducted by the National Center for Health Statistics using a complex, multistage probability sampling design. Data from the demographic, body measures, questionnaire, and examination components were linked using the unique participant identifier.

Participants aged 5–17 years were eligible for the present analysis because the seeing difficulty item (FNQ021) was administered only to this age group in the NHANES Functioning questionnaire. We first restricted the sample to participants aged 5–17 years, then excluded those with missing FNQ021 data. We subsequently excluded participants with missing BRI values or missing covariates included in the fully adjusted model. The final analytic sample comprised 1566 children and adolescents.

To assess the potential for selection bias related to the complete-case approach, we compared included participants with excluded age-eligible participants on age, sex, race/ethnicity, and poverty–income ratio (PIR) using survey-weighted methods. For this comparison, missing PIR values were retained as a separate category.

### 2.2. Assessment of VD

VD was assessed using NHANES item FNQ021, which asked whether the participant had difficulty seeing even when wearing glasses or contact lenses. The FNQ was administered as part of the household interview. Participants aged 16 years or older and emancipated minors were interviewed directly, whereas a proxy provided information for participants younger than 16 years and for those unable to answer themselves. Therefore, responses in the present study should be considered self- or proxy-reported depending on participant age and interview circumstances. Because proxy reporting was used for younger children, this measure may be affected by differences in reporting accuracy by age and interview circumstances. Response options included no difficulty, some difficulty, a lot of difficulty, and cannot do at all. Participants reporting some difficulty, a lot of difficulty, or inability to see were classified as having VD, whereas those reporting no difficulty were classified as non-VD. In a sensitivity analysis, we applied a stricter definition in which only participants reporting a lot of difficulty or inability to see were classified as having VD, while those reporting no difficulty or some difficulty were classified as non-VD.

### 2.3. Assessment of BRI

BRI was the primary exposure of interest. It was calculated from waist circumference and height using the following formula:


$$BRI=364.2-365.5\times \sqrt{1-(\frac{(\frac{WC}{2\pi }{)}^{2}}{(0.5\times Height{)}^{2}})}$$

Waist circumference and height were obtained from the NHANES body measures component and were expressed in the same unit. In the main analyses, BRI was examined both as a continuous variable and as a categorical variable based on quartiles, with the lowest quartile serving as the reference group.

### 2.4. Covariates

Covariates were selected a priori according to their potential relevance to both adiposity and visual health. Demographic characteristics included age, sex, and race/ethnicity, which was categorized as Non-Hispanic White, Mexican American, Non-Hispanic Black, Other Hispanic, and Other Race. Socioeconomic status was assessed using the PIR, which was further categorized as <1 and ≥1. Lifestyle-related variables included physical activity and daily screen time. Physical activity was assessed using the number of days in the past 7 days on which the participant had been physically active for at least 60 min per day. Participants reporting physical activity on 5 or more days per week were classified as active, whereas those reporting fewer than 5 days were classified as inactive. This cutoff was based on the 60 min/day pediatric physical activity recommendation and has been used as an operational threshold in population-based youth physical activity reporting [34]. Daily screen time was categorized as <2 h/day, 2–4 h/day, or >4 h/day, using the 2 h/day threshold commonly referenced in pediatric media-use guidance and separating higher screen exposure into additional categories [35]. In addition to the covariates included in the regression analyses, several anthropometric and early-life variables were summarized to further characterize the study population. These variables included waist circumference, height, body mass index (BMI), and birth weight.

### 2.5. Statistical Analysis

All analyses accounted for the complex sampling design of NHANES by incorporating the Mobile Examination Center (MEC) examination weight WTMEC2YR, the stratum variable SDMVSTRA, and the primary sampling unit variable SDMVPSU. The August 2021–August 2023 NHANES release represents a nationally representative 2-year cycle with an updated sample design and modified data collection procedures. Because our analysis combined questionnaire data (FNQ021) with examination data used to derive BRI (waist circumference and height), we used the MEC examination weight (WTMEC2YR) in accordance with Centers for Disease Control and Prevention (CDC) analytic guidance for analyses including both interview and examination variables. Continuous variables were presented as weighted means with standard deviations (SD), and categorical variables were expressed as unweighted counts with weighted percentages. Differences in baseline characteristics between participants with and without VD were evaluated using survey-weighted t tests for continuous variables and Rao–Scott adjusted chi-square tests for categorical variables. As an additional assessment of potential selection bias from complete-case exclusion, we compared included and excluded age-eligible participants on age, sex, race/ethnicity, and PIR using survey-weighted t tests for continuous variables and Rao–Scott adjusted chi-square tests for categorical variables. For this comparison, PIR was summarized with missing values retained as a separate category.

Survey-weighted logistic regression models were then fitted to examine the association between BRI and VD. Three models were fitted: Model 1 was unadjusted; Model 2 was adjusted for age, sex, and race/ethnicity; and Model 3 was further adjusted for PIR level, physical activity, and screen time. Odds ratios (ORs) and 95% confidence intervals (CIs) were reported.

To assess the association comprehensively, BRI was analyzed both as a continuous variable and in quartiles. To improve interpretability, we additionally expressed the association for continuous BRI per 1 SD increase by refitting the survey-weighted logistic regression models using a standardized BRI variable. For the quartile-based analysis, BRI quartiles were defined using survey-weighted cut-points derived from the weighted BRI distribution in the analytic sample. The weighted quartile cut-points were 2.15, 2.74, and 3.88. The lowest quartile was used as the reference group, and a *p* for trend was calculated by modeling the ordinal quartile variable as a continuous term. To further examine the dose–response relationship, restricted cubic spline (RCS) analysis was performed based on the fully adjusted survey-weighted logistic regression model using the ‘ns()’ function in the R ‘splines’ package, with two internal knots placed at 2.34 and 3.36. In addition, weighted VD prevalence by BRI quartile was summarized in Supplementary Tables to facilitate comparison between descriptive and modeled results. Overall and non-linear associations were evaluated to determine whether the relationship between BRI and VD deviated from linearity. Subgroup analyses were limited to two prespecified clinically relevant interactions, namely sex and age group. Interaction tests were performed to assess whether the association between BRI and VD differed across subgroups. These analyses were considered exploratory, and the results were interpreted cautiously in light of multiple comparisons and limited power for interaction testing. As a sensitivity analysis, the primary survey-weighted logistic regression models were repeated using a stricter definition of VD, in which only responses of “a lot of difficulty” or “cannot do at all” were classified as VD. All statistical analyses were conducted in R software (version 4.4.2; R Foundation for Statistical Computing, Vienna, Austria). Survey-weighted analyses were performed using the ‘survey’ package, with ‘svydesign()’ used to specify the complex sampling design and ‘svyglm()’ used for survey-weighted logistic regression. Survey-weighted cut-points were obtained using ‘wtd.quantile()’ from the ‘Hmisc’ package. RCS analyses were implemented using the ‘ns()’ function from the R ‘splines’ package. A two-sided *p* value < 0.05 was considered statistically significant.

## 3. Results

### 3.1. Participant Selection and Baseline Characteristics

**Figure 1:**
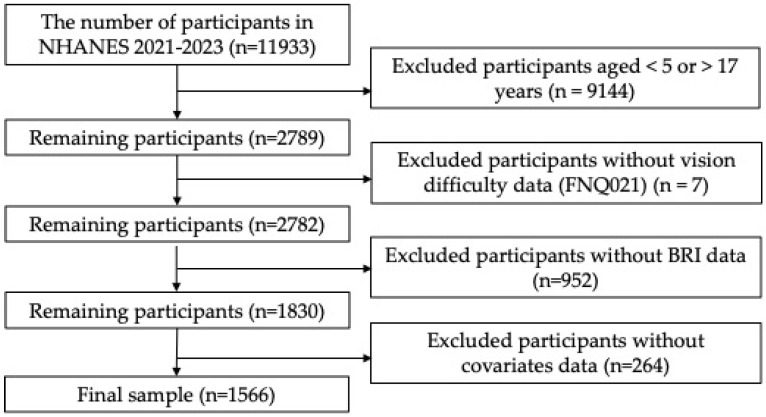
Flowchart of participant selection process. FNQ021 was administered only to participants aged 5–17 years.

As shown in Figure 1, 11,933 participants were initially identified from NHANES August 2021–August 2023. After restricting the sample to participants aged 5–17 years who were eligible for FNQ021, 2789 participants remained. We then excluded 7 participants with missing FNQ021 data, leaving 2782 participants with non-missing VD data. Of these, 952 were excluded because of missing BRI data, and 264 were excluded because of missing covariate data. The final analytic sample therefore consisted of 1566 children and adolescents, including 279 participants with VD and 1287 without VD. The unweighted prevalence of VD was 17.8% (279/1566), and the weighted prevalence was 16.2% (95% CI: 14.7–17.7%).

To evaluate the potential for selection bias introduced by complete-case exclusion, we compared included and excluded age-eligible participants on selected demographic characteristics (Supplementary Table S1). Included and excluded participants did not differ significantly in age (11.01 ± 3.70 vs. 11.20 ± 3.67 years, *p* = 0.504), but differed in sex (*p* = 0.007), race/ethnicity (*p* = 0.003), and PIR distribution (*p* < 0.001). These findings suggest that some degree of selection related to the complete-case approach cannot be excluded.

Baseline characteristics of the study population are presented in Table 1. Compared with participants without VD, those with VD were older (12.01 ± 3.50 vs. 10.81 ± 3.71 years, *p* < 0.001), were more likely to be female (54% vs. 46%, *p* = 0.02), and showed a different racial/ethnic distribution (*p* = 0.02). Participants with VD also had higher waist circumference (76.82 ± 17.52 vs. 71.01 ± 15.92 cm, *p* < 0.001), greater height (151.49 ± 18.34 vs. 146.71 ± 20.91 cm, *p* = 0.01), higher BMI (22.97 ± 7.33 vs. 20.57 ± 5.73, *p* < 0.001), and higher BRI (3.63 ± 1.89 vs. 3.17 ± 1.52, *p* = 0.004). In addition, a PIR level < 1 was more common among participants with VD (25% vs. 17%, *p* = 0.038), and longer daily screen time was also more frequent in the VD group (*p* = 0.004). No significant differences were observed in birth weight or physical activity between the two groups (all *p* > 0.05).

### 3.2. Association Between BRI and VD

The association between BRI and VD is shown in Table 2 and Table 3. When BRI was analyzed as a continuous variable, each one-unit increase in BRI was associated with higher odds of VD in all three models. The ORs (95% CIs) were 1.18 (1.08–1.29) in Model 1, 1.13 (1.03–1.23) in Model 2, and 1.12 (1.02–1.23) in Model 3, with corresponding *p* values of 0.001, 0.014, and 0.029, respectively. To improve interpretability, we additionally expressed the association per 1 SD increase in BRI. The weighted SD of BRI was 1.60. The ORs (95% CIs) per 1 SD increase in BRI were 1.30 (1.13–1.49) in Model 1, 1.21 (1.05–1.40) in Model 2, and 1.20 (1.03–1.40) in Model 3.

When BRI was categorized into weighted quartiles, the individual quartile estimates were not strictly monotonic, and the confidence intervals became wider in the fully adjusted model. Specifically, in Model 3, the ORs (95% CIs) for the second, third, and fourth quartiles were 0.95 (0.39–2.33), 1.23 (0.51–3.00), and 1.62 (0.66–4.00), respectively, compared with the lowest quartile. The weighted quartile cut-points were 2.15, 2.74, and 3.88, corresponding to Q1 ≤ 2.15, Q2 > 2.15 to ≤ 2.74, Q3 > 2.74 to ≤ 3.88, and Q4 > 3.88. Although the individual quartile estimates were imprecise and the fully adjusted trend test did not reach conventional statistical significance (*p* for trend = 0.066), the weighted prevalence of VD was higher in Q4 than in Q1 (22.6% vs. 13.8%; Supplementary Table S2), which was directionally consistent with the continuous and spline analyses.

In a sensitivity analysis using a stricter definition of VD, in which only participants reporting “a lot of difficulty” or “cannot do at all” were classified as cases, the association between BRI and VD remained positive. The ORs (95% CIs) for each one-unit increase in BRI were 1.42 (1.18–1.71) in Model 1, 1.35 (1.06–1.71) in Model 2, and 1.34 (0.97–1.86) in Model 3. Although the fully adjusted estimate became less precise under the stricter outcome definition, the direction of the association was consistent with that of the primary analysis. Detailed results are shown in Supplementary Table S3.

### 3.3. Dose–Response Relationship Between BRI and VD

**Figure 2:**
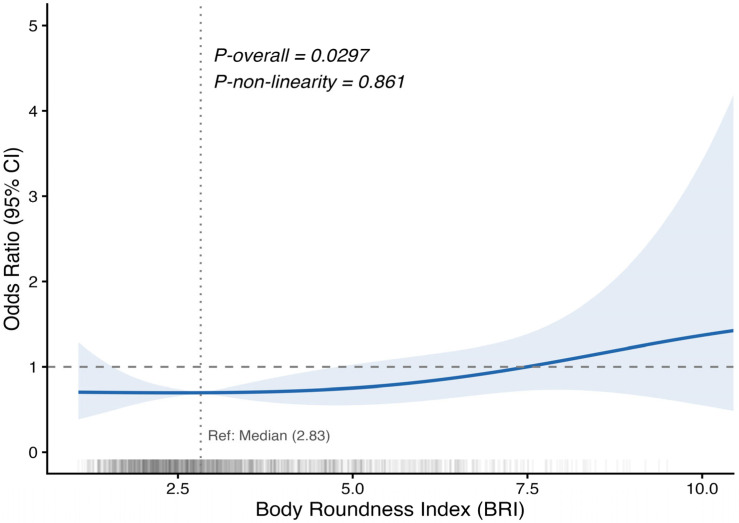
RCS analysis of the association between body roundness index and reported vision difficulty.

RCS analysis was performed to further characterize the dose–response relationship between BRI and VD (Figure 2). After adjustment for age, sex, race/ethnicity, PIR level, physical activity, and screen time, a significant overall association was observed between BRI and VD (*p*-overall = 0.0297). However, there was no evidence of a non-linear association (*p*-non-linearity = 0.861). The spline curve indicated that the odds of VD increased gradually with higher BRI values, supporting an approximately linear positive relationship across the observed range.

### 3.4. Subgroup Analyses

**Figure 3:**
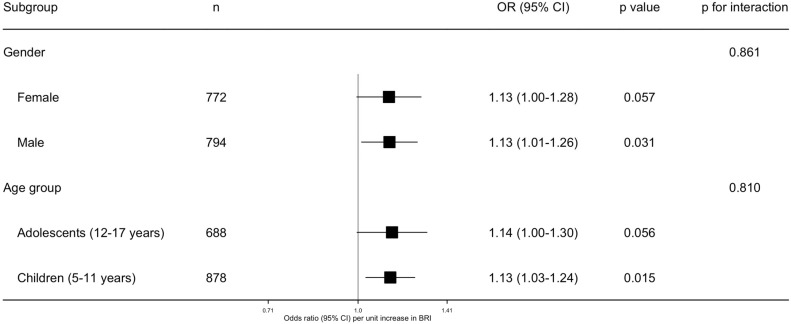
Subgroup analyses of the association between body roundness index and reported vision difficulty in prespecified strata defined by sex and age group. Age group was categorized as children aged 5–11 years and adolescents aged 12–17 years.

Subgroup analyses are presented in Figure 3. These exploratory analyses were limited to prespecified strata defined by sex and age group. No statistically significant interaction was detected for either sex (*p* for interaction = 0.861) or age group (*p* for interaction = 0.810).

## 4. Discussion

In this nationally representative cross-sectional study of U.S. children and adolescents aged 5–17 years, higher BRI was associated with greater odds of reported VD. This association remained after adjustment for demographic, socioeconomic, and lifestyle factors. When BRI was analyzed as a continuous variable, each one-unit increase in BRI was associated with 12% higher odds of VD in the fully adjusted model. RCS analysis suggested an approximately linear association across the observed range, whereas prespecified subgroup analyses by sex and age group did not show statistically significant interactions. Taken together, these findings suggest a modest population-level association between greater central adiposity, as reflected by BRI, and reported difficulty seeing in children and adolescents. Given the cross-sectional design and questionnaire-based outcome, the findings should be interpreted as exploratory and hypothesis-generating. Importantly, these findings should not be interpreted as evidence that obesity directly compromises visual function, but rather as an epidemiological association that may reflect residual confounding, shared behavioral pathways, reporting differences, or unmeasured ocular conditions.

Our findings add to a growing body of literature suggesting that adiposity may be relevant to pediatric vision-related outcomes. Previous studies have primarily focused on conventional obesity indicators, especially BMI, and have linked excess body weight to adverse ocular outcomes such as myopia, visual impairment, or structural retinal changes in children and adolescents [15,16,23,24,25,26,27]. However, evidence based on anthropometric measures that better capture body fat distribution remains limited. This distinction is important because central adiposity may be more metabolically active than generalized adiposity and may therefore be more closely related to biological processes relevant to ocular tissues [36,37]. Our study extends prior work by examining the association between BRI and reported functional visual difficulty in a nationally representative sample of U.S. children and adolescents.

Pediatric ophthalmic evidence may provide context for the observed association. Previous studies in children and adolescents with obesity have reported retinal vascular and choroidal alterations, including changes in retinal vessel caliber, retinal vascular geometry, choroidal thickness, and other retinal microvascular parameters [16,24,25,26,27]. These findings suggest that adiposity-related vascular or structural changes may be detectable early in life and provide a pediatric-specific rationale for examining whether BRI is associated with reported VD. More general metabolic pathways, including chronic low-grade inflammation, oxidative stress, insulin resistance, and endothelial dysfunction, may also be relevant because central adiposity is closely related to cardiometabolic and microvascular dysfunction [12,13,14,36,37,38,39,40,41]. However, because VD in the present study was assessed using a questionnaire item rather than objective ophthalmic testing, these pathways should be interpreted as biological context and hypotheses for future research rather than mechanisms demonstrated by the present analysis.

Behavioral and developmental pathways may also contribute. In childhood and adolescence, central adiposity often co-occurs with broader obesogenic behavior patterns, including sedentary behavior, poorer diet quality, and lower physical fitness [42,43,44]. Some of these same exposures, particularly greater screen time and less time spent outdoors, have also been implicated in adverse pediatric vision-related outcomes [45,46,47]. Although our models adjusted for physical activity and screen time, residual confounding by factors such as sleep characteristics, outdoor time, refractive correction, adequacy of eyeglass correction, and access to eye care cannot be excluded [48,49]. Reverse causation is also plausible. Children and adolescents with reported difficulty seeing may be less likely to participate in outdoor activities, organized sports, or other forms of physical activity and may spend more time in sedentary behaviors. Such behavioral changes could contribute to higher adiposity over time [50,51,52]. Therefore, the observed cross-sectional association may reflect bidirectional or shared behavioral pathways rather than a unidirectional effect of adiposity on vision-related complaints.

The RCS analysis indicated a broadly linear positive association between BRI and VD across the observed range, with little evidence of departure from linearity. This suggests that, within our study population, the association was not characterized by a clear threshold. When BRI was analyzed in quartiles, the estimates in the fully adjusted model became less precise, and the confidence interval for the highest quartile crossed 1.0. Although the weighted quartile estimates were imprecise and the fully adjusted trend test did not reach conventional statistical significance, the higher descriptive prevalence in Q4 compared with Q1, and the spline pattern remained directionally consistent with the positive continuous association. The quartile-based results likely reflect reduced precision after categorizing a continuous exposure and distributing 279 VD events across multiple categories. Therefore, the quartile-based results should be interpreted as exploratory and supportive rather than as the primary association estimates. Similarly, although the magnitude of the association varied somewhat across sex and age strata, neither interaction test was statistically significant. Because subgroup analyses were limited and exploratory, and because interaction testing may have been underpowered, these findings should be viewed cautiously.

This study has several strengths. It was based on NHANES data and therefore drew from a nationally representative sample of U.S. children and adolescents. The analysis accounted for the complex survey design and incorporated multiple clinically relevant covariates. We evaluated BRI as both a continuous variable and a categorical variable, and we further examined the shape of the association using RCS analysis. In addition, limited prespecified subgroup analyses were conducted according to sex and age group. These features strengthen the interpretability and robustness of the findings.

The use of NHANES data supports population-level inference for U.S. children and adolescents aged 5–17 years, an age range that spans important developmental transitions from childhood to adolescence. These stages differ in body composition, pubertal development, behavioral patterns, screen exposure, and visual demands, which may all be relevant to both adiposity and reported visual difficulty. At the same time, the findings should be generalized cautiously to populations outside the United States or to settings with different demographic compositions, obesity patterns, lifestyle environments, and access to pediatric eye care.

Several limitations should be acknowledged. First, because the study was cross-sectional, temporality could be established, and causal inference was not possible. Second, the outcome was based on reported VD rather than standardized ophthalmic examination, which may have introduced outcome misclassification and likely captured a heterogeneous range of visual problems rather than a single clinical entity. The FNQ021 item does not identify the cause, duration, or clinical basis of reported difficulty seeing and may reflect uncorrected or undercorrected refractive error, amblyopia, ocular disease, developmental factors, access-to-care disparities, or reporting bias. In addition, proxy reporting for younger children may have affected measurement validity across the 5–17-year age range. Third, although we adjusted for several major demographic and lifestyle variables, residual confounding from unmeasured factors remains possible, including outdoor time, sleep characteristics, refractive correction status, adequacy of eyeglass correction, and access to eye care. Fourth, the final analytic sample was obtained using a complete-case approach. Of 2789 age-eligible participants, 1566 were included in the final analysis, corresponding to approximately 56.2% of the eligible sample. Exclusions were mainly due to missing BRI values and missing covariates. Although we compared included and excluded age-eligible participants and found no significant difference in age, differences in sex, race/ethnicity, and PIR distribution suggest that selection bias cannot be excluded. Fifth, NHANES August 2021–August 2023 was the first public release including post-pandemic data collection and involved an updated sample design, modified data collection procedures, and lower response rates than earlier cycles; therefore, subgroup estimates may have been less precise and should be interpreted cautiously in light of CDC cycle-specific analytic guidance [53]. Finally, the present analysis did not investigate detailed metabolic biomarkers or mediation pathways. Several laboratory measures in NHANES, particularly fasting-related biomarkers, are available only in subsamples and require component-specific weights; therefore, they were not incorporated into the main descriptive or regression analyses. Consequently, this study cannot determine whether residual confounding, metabolic pathways, or specific ocular conditions explain the observed association.

Overall, this study found a modest positive association between BRI and reported VD in a nationally representative sample of U.S. children and adolescents. Because the outcome was questionnaire-reported and the study design was cross-sectional, these findings should be interpreted as exploratory and hypothesis-generating. Future longitudinal studies are needed to clarify temporality and potential bidirectional pathways. Such studies should include objective ophthalmic assessments, including best-corrected visual acuity, objective refraction, ocular diagnoses, and retinal or choroidal imaging, together with appropriately weighted metabolic biomarkers when available.

## 5. Conclusions

Higher BRI was modestly associated with greater odds of reported VD among U.S. children and adolescents. Given the cross-sectional design and questionnaire-based outcome, these findings should be interpreted as an exploratory population-level association rather than evidence of causality, clinical utility, or a direct effect of obesity on visual function.

## Figures and Tables

**Table 1:** Baseline characteristics of the study participants (NHANES 2021–2023).

Variables	Total *n* = 1566	Non-VD *n* = 1287	VD *n* = 279	*p* Value
Age (years)	11.01 ± 3.70	10.81 ± 3.71	12.01 ± 3.50	<0.001
Sex (%)				0.02
Female	772 (47)	610 (46)	162 (54)	
Male	794 (53)	677 (54)	117 (46)	
Race (%)				0.02
Non-Hispanic White	655 (49)	563 (51)	92 (37)	
Mexican American	212 (11)	167 (11)	45 (13)	
Non-Hispanic Black	219 (11)	170 (10.0)	49 (19)	
Other Hispanic	233 (12)	178 (11)	55 (17)	
Other Race	247 (16)	209 (17)	38 (14)	
Waist circumference (cm)	71.95 ± 16.33	71.01 ± 15.92	76.82 ± 17.52	<0.001
Height (cm)	147.48 ± 20.59	146.71 ± 20.91	151.49 ± 18.34	0.01
BMI (kg/m^2^)	20.96 ± 6.08	20.57 ± 5.73	22.97 ± 7.33	<0.001
BRI	3.24 ± 1.60	3.17 ± 1.52	3.63 ± 1.89	0.004
Birth Weight (kg)	3.26 ± 0.60	3.27 ± 0.58	3.22 ± 0.71	0.548
PIR level				0.038
<1	388 (19)	300 (17)	88 (25)	
≥1	1178 (81)	987 (83)	191 (75)	
Physical Activity (%)				0.469
Active	942 (60)	790 (60)	152 (57)	
Inactive	624 (40)	497 (40)	127 (43)	
Daily Screen time (%)				0.004
<2 h	339 (22)	303 (24)	36 (15)	
2–4 h	813 (51)	681 (52)	132 (50)	
>4 h	414 (26)	303 (25)	111 (36)	

Continuous variables are presented as weighted mean ± SD, and categorical variables as unweighted counts with weighted percentages. *p* values were calculated using survey-weighted *t* tests for continuous variables and Rao–Scott adjusted χ^2^ tests for categorical variables, accounting for the complex sampling design of NHANES.

**Table 2:** Association between weighted quartiles of BRI and VD using survey-weighted logistic regression (NHANES 2021–2023).

Model	Q1	Q2	Q3	Q4	*p* for Trend
Model 1	1	0.86 (0.56–1.33)	1.24 (0.77–1.99)	1.83 (1.14–2.95)	0.011
Model 2	1	0.96 (0.59–1.57)	1.26 (0.77–2.06)	1.67 (0.99–2.81)	0.031
Model 3	1	0.95 (0.39–2.33)	1.23 (0.51–3.00)	1.62 (0.66–4.00)	0.066

Model 1: unadjusted. Model 2: adjusted for age, sex, and race/ethnicity. Model 3: additionally adjusted for PIR level, physical activity, and screen time. All models accounted for the complex survey design of NHANES. BRI quartiles were defined using survey-weighted cut-points derived from the weighted BRI distribution. The cut-points were 2.15, 2.74, and 3.88.

**Table 3:** Association between continuous BRI and VD using survey-weighted logistic regression (NHANES 2021–2023).

Model	OR	95% CI	*p*
Model 1	1.18	1.08–1.29	0.001
Model 2	1.13	1.03–1.23	0.014
Model 3	1.12	1.02–1.23	0.029

Model 1: unadjusted. Model 2: adjusted for age, sex, and race/ethnicity. Model 3: additionally adjusted for PIR level, physical activity, and screen time. All models accounted for the complex survey design of NHANES.

## Data Availability

The data are publicly available online in CDC/National Center for Health Statistics (https://wwwn.cdc.gov/nchs/nhanes/Default.aspx; accessed on 1 March 2026).

## Supplementary Materials

The following supporting information can be downloaded at: https://www.mdpi.com/article/10.3390/healthcare14101352/s1, Table S1: Comparison of included and excluded age-eligible participants under the complete-case approach; Table S2: Weighted prevalence of reported vision difficulty by weighted BRI quartile; Table S3: Sensitivity analysis of the association between BRI and reported vision difficulty using a stricter outcome definition.

## Abbreviations

The following abbreviations are used in this manuscript:

BMI	Body mass index
BRI	Body roundness index
CI	Confidence interval
NHANES	National Health and Nutrition Examination Survey
OR	Odds ratio
PIR	Poverty–income ratio
RCS	Restricted cubic spline
VD	Vision difficulty
