# Supplementary material for: Body Roundness Index and Reported Vision Difficulty in U.S. Children and Adolescents: A Cross-Sectional Study of NHANES 2021–2023
Source: Healthcare (Basel). 2026 May 15;14(10):1352. doi: 10.3390/healthcare14101352 (PMC13206060; doi:10.3390/healthcare14101352)
Supplement: Supplementary file 1 [file healthcare-14-01352-s001.zip › healthcare-4235374-supplementary.pdf]

Supplementary Table S1. Comparison of included and excluded age-eligible participants under the complete-case approach.

Variables	Included<br>*n* = 1566	Excluded<br>*n* = 1223	P
Age (years)	11.01 ± 3.70	11.20 ± 3.67	0 504
Sex			0 007
Female	772 (47)	623 (57)	
Male	794 (53)	600 (43)	
Race/ethnicity			0 003
Non-Hispanic White	655 (49)	444 (30)	
Mexican American	212 (11)	185 (17)	
Non-Hispanic Black	219 (11)	203 (16)	
Other Hispanic	233 (12)	172 (17)	
Other Race	247 (16)	219 (19)	
PIR level			<0.001
<1	388 (19)	204 (7.7)	
>=1	1,178 (81)	550 (18)	
Missing	0 (0)	469 (75)	

Supplementary Table S2. Weighted prevalence of reported vision difficulty by weighted BRI quartile

Quartile	BRI range	Unweighted n	Weighted VD prevalence, % (95% CI)
Q1	≤ 2.15	366	13.8% (10.4%–17.2%)
Q2	> 2.15 to ≤ 2.74	375	12.1% (7.8%–16.3%)
Q3	> 2.74 to ≤ 3.88	402	16.5% (13.0%–20.0%)
Q4	> 3.88	423	22.6% (17.7%–27.6%)

Supplementary Table S3. Sensitivity analysis of the association between BRI and reported vision difficulty using a stricter outcome definition.

Model	OR	95% CI	P
Model 1	1 42	1.18–1.71	0 002
Model 2	1 35	1.06–1.71	0 039
Model 3	1 34	0.97–1.86	0 063

Model 1: unadjusted. Model 2: adjusted for age, sex, and race/ethnicity. Model 3: additionally adjusted for PIR level, physical activity, and screen time. All models accounted for the complex survey design of NHANES.
